# A Questionnaire-Based Cross-Sectional Survey of Knowledge, Attitudes, and Practices toward COVID-19 among Students and Staff in Asir, Saudi Arabia during the Second Wave of the Pandemic

**DOI:** 10.3390/vaccines10122014

**Published:** 2022-11-25

**Authors:** Norhan Saif Sheraba, Khalid Orayj, Ali Alqahtani, Rehab H. El-sokkary, Mohammad Khalid

**Affiliations:** 1 Department of Pharmaceutics, College of Pharmacy, King Khalid University, Asir-Abha 61421, Saudi Arabia; 2 VACSERA, The Holding Company for Biological Products and Vaccines, Giza 12311, Egypt; 3 Department of Clinical Pharmacy, College of Pharmacy, King Khalid University, Asir-Abha 61421, Saudi Arabia; 4 Department of Pharmacology, College of Pharmacy, King Khalid University, Asir-Abha 61421, Saudi Arabia; 5 Medical Microbiology and Immunology, Faculty of Medicine, Zagazig University, Zagazig 44519, Egypt

**Keywords:** COVID-19, KAP, cross-sectional survey, Saudi Arabia

## Abstract

Being in a rapidly changing and dynamic environment during the COVID-19 pandemic, individuals’ perceptions change on a daily basis, and this starts to inform the status of knowledge, attitude, and practices (KAP) during the second wave of the pandemic. Aim: To assess the KAP on COVID-19 among students, teaching staff, and administrative staff in the Asir region for the first time. Methods: A questionnaire-based survey cross-sectional study was conducted from 10 February to 10 March 2021 using an online questionnaire. Results: In the survey of students and staff in educational institutes in the Asir region, the target population were well informed about COVID-19; in 10 out of 13 questions, more than 80% of the respondents answered correctly. The attitude of the target populations was quite positive. They were worried about the increase in the number of COVID-19 cases. The target populations were concerned (94.8%) for their kith and kin not to get infected. The target populations agree with the precautionary measures that were taken by Saudi authorities. A positive association was found between knowledge and practices as well as knowledge and attitudes of the target population. A significantly positive association was observed between attitudes and practices of the educational institutes’ respondents in Asir, Saudi Arabia. Conclusion: The students and staff in educational institutes in the Asir region have a high level of knowledge about COVID-19, have a positive attitude toward it, take proper precautions against it, and are enthusiastic about the COVID-19 vaccine.

## 1. Introduction

The SARS-CoV-2 outbreak first started in November and was reported in December 2019 in Wuhan, China [1]. The virus is highly transmissible, mainly through respiratory routes, spreading across the globe and so the WHO declared COVID-19 as a pandemic on 11 March 2020 [2], which led to the start of public lockdowns across the globe.

So far, we do not have specific treatments for COVID-19. The available vaccines are significantly contributing to controlling the viral spread as well as decreasing the severity of COVID-19, but vaccines are also associated with complications [3]. A significant number of infection cases have been reported even in the vaccinated individuals [4]. The occasional advent of various viral variants further compounded the containment of the COVID-19 pandemic. The respiratory route of the viral transmission makes it more lethal, as air inhalation cannot be escaped.

The COVID-19 pandemic has been linked to overcrowding and poor infection control practices. SARS-CoV-2 spread and the COVID-19 pandemic has been controlled very well in Saudi Arabia by implementing very strong control measures [5]. The Kingdom of Saudi Arabia (KSA) moved swiftly to enact ground-breaking steps to limit the transmission; on 27 February 2022, the KSA imposed restrictions on the inbound Umrah pilgrimage. The Kingdom also restricted the movement of Gulf Cooperation Council (GCC) individuals who had recently visited COVID-19-impacted countries, terminated the then recently implemented e-VISA program, and prohibited people from entering from COVID-19 affected nations [6]. Finally, the Kingdom’s universities, colleges, and mosques were all closed [7,8]. Although the infection control measures that were imposed by the government and the Ministry of Health (MOH) were the primary intervention to minimize the spread of the virus in both healthcare settings and to the community, a great role was still available for the level of awareness and response in the community [9,10,11,12], especially after the availability of vaccines. At that time, many studies suggested that vaccine hesitancy is affected by the knowledge of a population about virus prevention [13,14]. Late during the pandemic, many KAP studies were published about COVID-19. However, the rapidly changing and dynamic environment, where individuals’ perceptions change on a daily basis according to the pandemic status and morbidity and mortality rates across the country, highlighted the need to gain information about the status of KAP during the second wave of pandemic. Hence, in the present study we aimed to assess the knowledge, attitudes, and practices (KAP) on COVID-19 among students, teaching staff, and administrative staff in the Asir region. The advocate role of this target population to promote the proper knowledge to the community is indispensable, in addition to being a role model in the proper application of preventive measures. To the best of our knowledge, this is the first study to present such data from the Asir region. As geographical distribution and social determinants are important factors in the development of proper prevention plans, we believe this study will help us to understand some essential aspects for controlling the current or any subsequent pandemics in this region.

## 2. Methodology

### 2.1. Study Design, Setting, and Population

This survey was conducted as an observational cross-sectional study using a pre-validated questionnaire from 10 February to 10 March 2021, for a four-week duration. An online questionnaire was prepared using a Google form and distributed through social media using a snowball technique among students, teaching staff, and administrative staff from the Asir Region of Saudi Arabia. The questionnaire was originally made in English then translated into Arabic.

### 2.2. Study Population and Sample Size

In the Asir area, there are 544,251 students, teaching, and non-teaching staff in educational institutions [15,16]. The nonprobability convenience sampling approach was used to select the respondents. The estimated minimum target sample size was 384, which was measured with a confidence interval of 95%, an acceptable margin of error of 5%, an expected frequency of 50%, and a design effect of 1.0 through STATCALC on Epi Info software. The inclusion criteria were as follows: Saudi/non-Saudi students, faculty members, and administrative staff in the Asir Region. The participants who refused to participate were excluded from the study [17].

### 2.3. Survey Design and Pilot Study

After a review of the literature [18,19,20], the authors prepared a standardized questionnaire in English. The draft questionnaire was validated by five academicians for review and comments in order to ensure applicability, suitability, and consistency in our environment, as well as to define the face and content validity. It was piloted by 15 persons for feedback, remarks, and advice on its transparency, simplicity, clarity of questions, and comprehension. The answers of the pilot study were not included in the final analysis, but the input was reviewed, the double-barreled, confusing, and leading questions were corrected, and a finalized form of the questionnaire was developed.

### 2.4. Data Collection

The survey was carried out using an online Google Form to make it easier for the participants to fill out the questionnaire. The survey was divided into four sections. Section 1 included items about the participants’ background and sociodemographic characteristics. Age, gender, nationality, race, place of residence, and academic program were all identified. Section 2 consisted of 13 items and was designed to assess the target population’s understanding of COVID-19. “Yes”, “no”, and “I don’t know” were the answers to the questions on the signs and symptoms of coronavirus, as well as preventative steps and advice.

Section 3 included eight items to assess the respondent’s attitude towards COVID-19, and Section 4 looked at how the respondents handled the latest epidemic. It consisted of seven questions, with three possible answers from the participants: “Accept,” “Disagree,” and “Neutral”. The respondents’ awareness of, attitude against, and precautionary measures toward COVID-19 were the dependent variables in this analysis. The age, ethnicity, nationality, education levels, and comorbid conditions of the respondents served as independent variables in this analysis. The knowledge, attitude, and practice (KAP) scores for each participant were determined after the answers were tallied [21].

The initial Bloom’s cut-off points of 80.0–100.0%, 60.0–79.0%, and 59.0% were adapted and updated from a KAP study on dengue fever prevention in Male, Maldives, and Bangkok in 2007 and a KAP study on COVID-19 among chronic disease patients in Northwest Ethiopia in 2020 [22].

For the knowledge section (13 questions, total score: 13), each correct answer (yes) received a score of one, while incorrect responses (no and I don’t know) received a score of zero for the information portion. Bloom’s cut-off point was used to categorize participants’ total knowledge scores, which were classified as high between 11 and 13 points, moderate between 8 and 10 points, and low below 7 points. The overall attitude score (8 questions, total score: 8) was classified as positive if it was between 6.4 and 8 points, neutral if it was between 4.8 and 6.3 points, and negative if it was less than 4.7 points, using the same Bloom’s cut-off point.

For the precautionary measures section (7 questions, total score: 7), yes was assigned a score of 1, sometimes a score of 0, and no a score of 0. The overall score was graded as acceptable for a score between 5.6 and 7 points, mild for a score between 4.2 and 5.5 points, and unacceptable for a score of less than 4.2 points using the same Bloom’s cut-off point.

### 2.5. Statistical Analysis

The responses were collected in Microsoft Excel spreadsheets (2016). The downloaded data were analyzed using SPSS 21.0 statistical software (IBM Inc., Chicago, IL, USA). The results were described in terms of mean frequencies or percentage for descriptive statistics. Significance for comparison was performed by the Mann–Whitney U test to compare two groups. The Kruskal–Wallis test was performed to compare more than two groups in the survey. For association analysis, Spearman correlations were used.

### 2.6. Ethical Approval

The participants’ inclusion in the questionnaire was entirely voluntary. The data confidentiality was maintained. After reading the introductory questionnaire consent form, which outlined the study’s goals and conditions, the participants were asked to choose an alternative (Yes/No) as informed written consent before answering the questionnaire. Ethical approval was obtained from the Research Ethics Committee at King Khalid University (HAPO-06-B-001), on 24 December 2020, with an approval number (ECM#2020-3205)-(HAPO-06-B-001).

## 3. Results

### 3.1. Demography of Target Population

A total of 2191 responses were analyzed. The highest proportion of respondents (38.6%) belonged to the 20–30 age group. These responses were mainly from students (51.4%) with bachelor education level (68.6%) and more than two-thirds of respondents were female, which contributed 70.5%. A total of 76.8% of respondents in the study were from people living in the urban area of Asir. More than two thirds of the respondents (72.8%) had attended COVID-19 infection training or orientation sessions. Only 16% of the respondents had been infected with the SARS-CoV-2 but 71.3% of respondents had encountered COVID-19 patients closely. Although all forms of the media contributed to informing people about the COVID-19, the target population mostly relied on the Saudi government websites and media to get informed about the pandemic (Table 1). Supplementary Table S1 shows the results of Kruskal–Wallis and Mann–Whitney tests to examine the association between knowledge, attitude, practice, and other factors. Its shows that there were significant differences in KAP among different ages, gender, employments status, places of residence, and levels of education (Supplementary Table S1).

### 3.2. Knowledge of the Target Population towards COVID-19

Table 2 presents the respondents who were well informed about the COVID-19; in 10 out of 13 questions, more than 80% of respondents answered correctly. The respondents in Asir showed a good understanding of the importance of preventive measures, such as wearing masks and maintaining social distancing to contain COIVD-19. This is shown in the response of the questions related to preventive measures where more than 90% of respondents correctly answered these questions.

### 3.3. Attitudes of the Target Population towards COVID-19

More than 75% of people responded positively to six questions. They were worried by the surge of the COVID-19 cases. A total of 94.8% of the target population were concerned for their kith and kin not to get infected rather than themselves. The target populations agree (89%) with the precautionary measures that were taken by Saudi authorities and supported (93%) the postponing or cancelling of mass gatherings (Table 3).

### 3.4. Practices of the Target Population towards COVID-19

More than 90% respondents washed their hands for more than 20 s, and over 93% of the respondents wash hands after touching the personal items of a person with a cough or cold. The target population washed their hands (89.3%) after touching grocery bags, while 86.3% of the respondents washed their hands after touching doorknobs. The use of gel sanitizer was practiced by 91.9% of the target population. A total of 86.1% of the respondents avoided touching their face, eyes, nose, and mouth and 88.3% wore face mask outside the home. Frequently used gadgets were disinfected by 72.8% of the respondents (Table 4).

### 3.5. Association Analysis of the Target Population towards COVID-19

**Figure 1:**
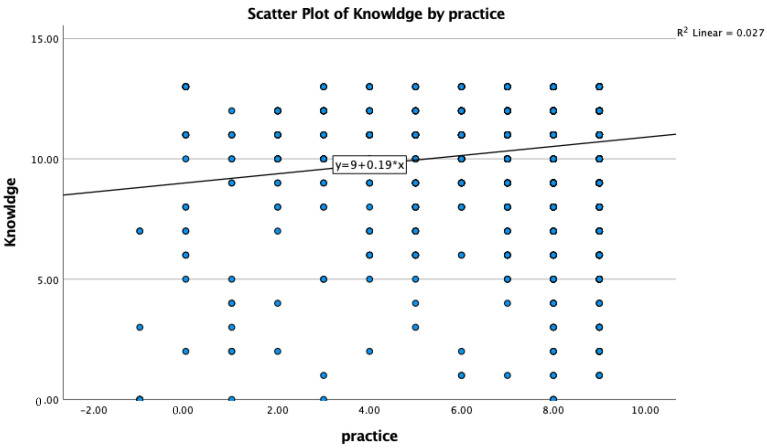
Knowledge and practice of the surveyed population a showed negligible positive association.

**Figure 2:**
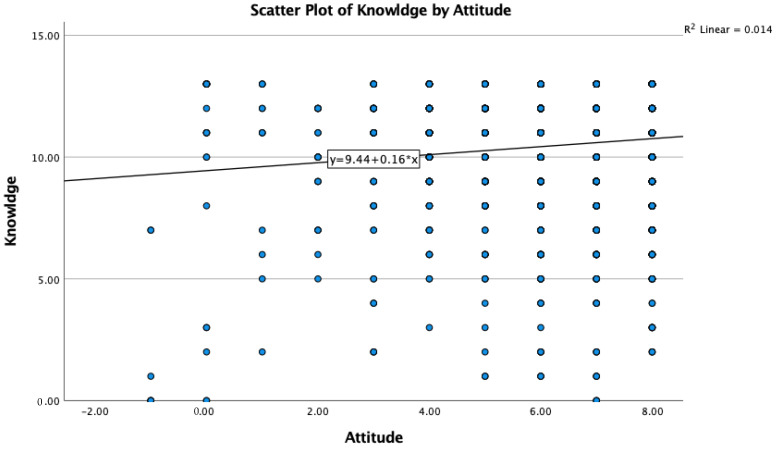
Knowledge and attitude of the target population also showed a negligible positive association.

**Figure 3:**
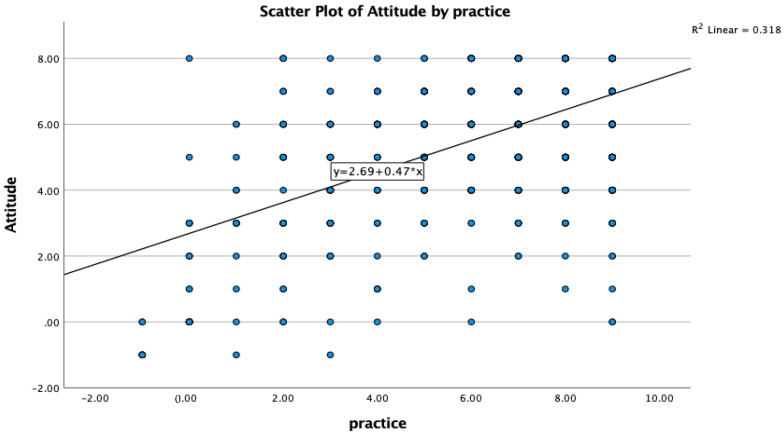
A positive association was observed between the attitude and practice of the surveyed population.

The Spearman two-tailed correlation analysis between the knowledge and practices of the target population showed a negligible positive association with a correlation coefficient of 0.07 (Figure 1, Table 5). Similarly, knowledge and attitude of the target population also showed negligible positive association with a correlation coefficient value of 0.05 (Figure 2, Table 6). Interestingly, a moderate positive association was observed between attitude and practice of the respondents with a correlation coefficient of 0.367 in Asir (Figure 3, Table 7).

## 4. Discussion

Since its start in November 2019, COVID-19 has severely affected the world and its death toll and financial damages seemed to be innumerable. COVID-19 has had more impact on some countries than on others. One of the worst examples is the Hajj and Umrah pilgrimage in Saudi Arabia, with both open only to a limited number of people with compulsory vaccination and ensured precautionary measures. The COVID-19 vaccine significantly helps people in protection from the disease, but even double vaccinated people are still prone to SARS-CoV-2 infection [3,4]. Mutations in the viral genome keep producing new variants with a recent viral variant named Omicron becoming the dominant variant of the virus in many western European countries where a double vaccination rate is more than 70% [23]. As such, the preventive measures are still the mainstay to avoid SARS-CoV-2 infection and thus, the COVID-19 pandemic.

Many studies [21,24] have been published to investigate the KAP in the Asir region, yet to the best of our knowledge, this is the first one in the region surveying people that are associated with educational institutes to a larger scale, which are considered as a source of knowledge and associated people are considered as ambassadors of that knowledge. As such, we decided to perform a KAP survey on COVID-19 from students, teaching, and non-teaching staff in the Asir region of Saudi Arabia.

Based on the population in educational institutes in Asir, the Epi Info software program required 381 survey responses to have a significant study but our target population was very keen to respond to the KAP survey on COVID-19 and we have got 2191 responses. The other published surveys in the region analyzed 740 participants [21], and asserted that the population of Asir is 2.26 billion, which is not true. Another study was based on 178 participants only [24], which is less than is required to have meaningful analysis. In the survey, we have covered a variety of age groups, ranging from below 18 years all the way up to above 60 years of age. In contrast, the Adam et al. study had almost 90% of the participants belonging to the 22–25 years age group, which makes our survey very informative to hear from all different age groups and helped us to draw a more complete picture of the people living in the Asir region.

Students were the highest respondents (51.4%) and that reflects in the age group also, as most of the respondents (38.6%) belonged to 20–30 years of age, which was also found in another study [24]. Interestingly, more than two-thirds of the respondents were female (70.5%), suggesting the females were keener to respond to the KAP survey on COVID-19. Higher female participation (88.5%) was also found in another study from the Asir region [21]. We got more than three-quarters (76.8%) of the responses from the target population who lived in urban areas as they were more exposed to the media and awareness programs that are run by the government. The study was designed to have a survey on the population belonging to educational institutes and the majority of the respondents (68.8%) were educated at a bachelor level. A similar result (60.1%) was also reported in the Adam et al. 2021 study, which is obvious from the previous assertions as most of the respondents belonged to 20–30 years of age and were students. Interestingly, a significant number of the respondents (72.8%) attended a COVID-19 infection training or orientation session, which suggests that the target population was keen to know the infectious agent, transmission, symptoms, and preventive measures of COVID-19. The survey revealed that 16% of the respondents were infected with SARS-CoV-2 but a significant number (71.3%) of their acquaintances had encountered COVID-19. Remarkably, the majority of the population was interested in taking the COVID-19 vaccine (56%), which was also found in the neighboring Jizan region survey [22], although many other studies reported hesitancy in vaccination of surveyed people [25,26,27,28,29].

This high degree of awareness might be attributed to the Saudi Ministry of Health’s attempts to raise public awareness of this unique virus by sending text messages and using other forms of communication, such as television and social media. Furthermore, we discovered a substantial difference in the knowledge level across individuals with various educational levels; those with a lower educational level had less knowledge than those with a higher educational level. Other studies also found a high level of knowledge among participants of the survey but they did not find significant variations in knowledge among various groups [21,24].

The attitudes of the target populations were quite positive, which has been found repeatedly in many studies [21,24,30,31,32]. They were worried about a surge in the number of COVID-19 cases. Interestingly, the survey revealed that the target populations were more concerned (94.8%) for their kith and kin not to get infected than themselves, which was also reported in Khaled et al. 2020 [21] survey. The target populations showed good faith in the Saudi government and agreed with the precautionary measures that were taken by Saudi authorities. They practiced most of the precautionary measures that were recommended by the Saudi authorities. These responses suggest that target populations were very responsive towards precautionary measures that were taken by the Saudi authorities, which helped them to curb COVID-19.

A similar study was conducted by Khaled et al. 2020 on the knowledge and attitude of the community from the Asir region, Saudi Arabia, toward COVID-19 and their precautionary measures against the disease. This study confirms that the residents of the Asir Region have a high knowledge of, demonstrate a positive attitude toward, and use appropriate precautionary measures against COVID-19, which is associated with obtaining information about the virus. This is in contrast to our study where we found that good practices are better associated with attitude rather knowledge about the COIVD-19.

It is worth noting that in this study, better COVID-19 knowledge scores were shown to be connected with a decreased risk of unfavorable attitudes and potentially harmful behaviors regarding the COVID-19 epidemic. These findings highlight the necessity of enhancing COVID-19 understanding among students, teaching staff, and non-teaching staff through health education, which may lead to changes in their attitudes and behaviors concerning COVID-19.

Interestingly, the association analysis in the present survey on KAP revealed that the knowledge of the target population about COVID-19 was very weakly associated with practice but the attitude of the surveyed population was associated with practice to contain the pandemic, which suggest that attitude is a bigger factor to move people to practice measures correctly to curb the COVID-19.

## 5. Conclusions

The KAP survey that we performed in the Asir region revealed that the target population has good knowledge about COIVD-19. They understood well and exhibited a positive attitude towards the importance of the precautionary measures, such as wearing a mask, maintaining social distancing, and washing their hands for more than 20 s after touching grocery bags, doorknobs, and personal items of a person with cough or cold. This is the consequence of the efforts that were made by the Saudi authorities. The people are more concerned for their kith and kin not to be infected by SARS-CoV-2 than themselves. The target population is quite satisfied with the measures that were taken by the Saudi government to curb the pandemic and the majority of them are willing to take the vaccine.

## 6. Limitations of the Study

Being an online questionnaire exposes the study to selection and recall bias possibilities. For ease of filling out the form at a time of being overwhelmed by COVID-19, we used a “yes/no” option. The concern of a higher knowledge scores can’t be ruled out.

## Figures and Tables

**Table 1:** Demographics of the survey population.

Demographic	*n* (%) N = 2191
Age group	
Under 18 years old	233 (10.6)
20–<30 years old	845 (38.6)
30–<40 years old	436 (19.9)
40–<50 years old	513 (23.4)
50–60 years old	143 (6.5)
>60 years	21 (1)
Gender (female)	1544 (70.5)
Status	
Administrative Staff	364 (16.6)
Student	1127 (51.4)
Teaching Staff	700 (31.9)
Place of residence	
Rural	509 (23.2)
Urban	1682 (76.8)
Level of education	
Bachelor	1508 (68.8)
Master	85 (3.9)
Others	544 (24.8)
PhD	54 (2.5)
Have you ever attended COVID-19 infection training or orientation session? (yes)	1594 (72.8)
Have you been infected with COVID-19? (yes)	351 (16)
Has any of your colleagues been infected with COVID-19? (yes)	1562 (71.3)
Would you like to take COVID-19 vaccine? (yes)	1226 (56)

**Table 2:** Knowledge of the survey population about COVID-19.

Knowledge about COVID-19	Yes * n (%)	No *n* (%)	I Don’t Know *n* (%)
1. The major clinical signs of COVID-19 are fever, fatigue, dry cough, and muscle aches.	1859 (84.8)	67 (3.1)	265 (12.1)
2. Stuffy nose, runny nose, and sneezing are less common in COVID-19 than in common cold.	1292 (59)	293 (13.4)	606 (27.7)
3. Alarming signs for COVID-19 that necessitate immediate medical care include difficulty of breathing, persistent pain or pressure in the chest.	1944 (88.7)	32 (1.5)	215 (9.8)
4. COVID-19 symptoms may appear 2–14 days after exposure to a source of SARS-CoV-2 virus.	1738 (79.3)	115 (5.2)	338 (15.4)
5. Not all persons with COVID-19 will progress from mild case to severe one.	865 (39.5)	698 (31.9)	628 (28.7)
6. COVID-19 may be transmitted to others even if the infected person is asymptomatic (Show no symptoms)	1846 (84.3)	97 (4.4)	248 (11.3)
7. The COVID-19 is transmitted through respiratory droplets of infected individuals.	1935 (88.3)	74 (3.4)	182 (8.3)
8. There is currently no specific treatment for COVID-19.	1355 (61.8)	387 (17.7)	449 (20.5)
9. Early detection and supportive treatment are corner stone in COVID-19 management.	1816 (82.9)	90 (4.1)	285 (13)
10. Persons who wear protective masks help control the spread of COVID-19.	2001 (91.3)	111 (5.1)	79 (3.6)
11. For not to get infected by the COVID-19 virus, people should avoid visiting crowded places.	2124 (96.9)	16 (0.7)	51 (2.3)
12. Physical distancing and treatment of COVID-19 cases are effective ways to minimize the spread of the virus.	2124 (96.9)	67 (3.1)	0 (0)
13. People who have connected with infected COVID-19 individuals should immediately follow the self-quarantine procedures for 14 days.	2086 (95.2)	49 (2.2)	56 (2.6)

Note: every question holds 1 mark, so the total score in the knowledge section is 13, which was used in the Mann–Whitney test and Kruskal–Wallis test for association analysis. * The correct answer in all questions is “yes”.

**Table 3:** Attitude of the survey population towards COVID-19.

Attitude towards COVID-19	Agree * n (%)	Disagree *n* (%)	Neutral *n* (%)
1. I am always ready to protect myself from getting infected by the SARS-CoV-2 virus.	1901 (86.8)	182 (8.3)	108 (4.9)
2. I think it is necessary to store enough amount of food, and medicines to avoid going out of the house during the quarantine period	1454 (66.4)	443 (20.2)	294 (13.4)
3. I am worried to get infected by SARS-CoV-2 virus.	1195 (54.5)	687 (31.4)	309 (14.1)
4. It is necessary to take actions to prevent the possible transfer of COVID-19 to my family members.	2076 (94.8)	66 (3)	49 (2.2)
5. I feel anxious, when hearing news about increasing number of patients infected with COVID-19.	1717 (78.4)	300 (13.7)	174 (7.9)
6. I believe that COVID-19 will be successfully controlled soon.	1685 (76.9)	274 (12.5)	232 (10.6)
7. I support the government-compulsory obligatory home quarantine for up to 2 weeks for people who have been close with someone who has been examined positive for COVID19.	1952 (89.1)	123 (5.6)	116 (5.3)
8. I support postponing or cancelling mass gatherings such as festivals, or sporting events.	2044 (93.3)	79 (3.6)	68 (3.1)

* Every question holds 1 mark, so the total score in the attitude section is 8, which was used in Mann–Whitney test and Kruskal–Wallis test for association analysis.

**Table 4:** Practices performed by the survey population to contain COVID-19.

Questions about Practices to Control COVID-19	Agree * n (%)	Disagree *n* (%)	Neutral *n* (%)
1. Essential proper hygiene begins with washing hands using soap for 20 s.	1989 (90.8)	125 (5.7)	77 (3.5)
2. I wash my hands after touching personal items of someone who has cough and/or cold	2052 (93.7)	77 (3.5)	62 (2.8)
3. I wash my hands after touching any plastic or paper from sources such as groceries.	1956 (89.3)	131 (6)	104 (4.7)
4. I wash my hands after touching doorknobs.	1891 (86.3)	158 (7.2)	142 (6.5)
5. I eat nutritious foods such as vegetables and fruits to keep healthy.	1936 (88.4)	103 (4.7)	152 (6.9)
6. I use hand gel to sanitize my hands.	2013 (91.9)	98 (4.5)	80 (3.7)
7. I disinfect cell phones, headsets, laptops, and all other gadgets that are frequently used.	1595 (72.8)	365 (16.7)	231 (10.5)
8. I avoid touching the face, eyes, nose and mouth.	1886 (86.1)	167 (7.6)	138 (6.3)
9. I wear a facemask when leaving home.	1935 (88.3)	142 (6.5)	114 (5.2)

* Every question holds 1 mark, so the total score in the practice section is 9, which was used in Mann–Whitney test and Kruskal–Wallis test for association analysis.

**Table 5:** Association of the Spearman correlation between knowledge and practice.

Correlations				
			Practice	Knowledge
Spearman’s rho	Practice	Correlation Coefficient	1	0.070 **
		Sig. (2-tailed)	.	0.001
		N	2124	2124
	Knowledge	Correlation Coefficient	0.070 **	1
		Sig. (2-tailed)	0.001	.
		N	2124	2191

** Correlation is significant at the 0.01 level (2-tailed).

**Table 6:** Association of the Spearman correlation between knowledge and attitude.

Correlations				
			knowledge	attitude
Spearman’s rho	Knowledge	Correlation Coefficient	1	0.050 *
		Sig. (2-tailed)	.	0.018
		N	2191	2191
	Attitude	Correlation Coefficient	0.050 *	1
		Sig. (2-tailed)	0.018	.
		N	2191	2191

* Correlation is significant at the 0.05 level (2-tailed).

**Table 7:** Association of the Spearman correlation between attitude and practice.

Correlations				
			Attitude	Practice
Spearman’s rho	Attitude	Correlation Coefficient	1	0.367 **
		Sig. (2-tailed)	.	0
		N	2191	2124
	Practice	Correlation Coefficient	0.367 **	1
		Sig. (2-tailed)	0	.
		N	2124	2124

** Correlation is significant at the 0.01 level (2-tailed).

## Data Availability

Not applicable.

## Supplementary Materials

The following supporting information can be downloaded at: https://www.mdpi.com/article/10.3390/vaccines10122014/s1, Table S1: Survey Statistics.
