# Supplementary material for: A Questionnaire-Based Cross-Sectional Survey of Knowledge, Attitudes, and Practices toward COVID-19 among Students and Staff in Asir, Saudi Arabia during the Second Wave of the Pandemic
Source: Vaccines (Basel). 2022 Nov 25;10(12):2014. doi: 10.3390/vaccines10122014 (PMC9783384; doi:10.3390/vaccines10122014)
Supplement: Supplementary file 1 [file vaccines-10-02014-s001.zip › vaccines-1931526-supplementary.pdf]

**Supplementary Table S1: Survey Statistics**

| | Knowledge (Total score =13) | | Attitude (Total score=8) | | Practice (Total score=9) | |
|---|---|---|---|---|---|---|
| **Demographics** | **P value*** | **Direction of association** | **P value** | **Direction of association** | **P value** | **Direction of association** |
| **Age group**** | 0.000 | | 0.000 | | 0.000 | |
| **Median (knowledge, attitude, practice)** | 0.000 | Under 18 years old is lower than 20 - < 30 years old | 0.000 | 20 - < 30 years old is lower than 30 - < 40 years old | 0.000 | Under 18 years old is lower than 30 - < 40 years old |
| | 0.000 | Under 18 years old is lower than 50 - 60 years old | 0.000 | 20 - < 30 years old is lower than 40 - < 50 years old | 0.000 | Under 18 years old is lower than 40 - < 50 years old |
| | 0.000 | Under 18 years old is lower than 30 - < 40 years old | 0.017 | under 18 years old is lower than 30 - < 40 years old | 0.000 | Under 18 years old is lower than 50 - 60 years old |
| | 0.000 | Under 18 years old is lower than 40 - < 50 years old | 0.009 | under 18 years old is lower than 40 - < 50 years old | 0.000 | 20 - < 30 years old is lower than 30 - < 40 years old |
| | | | | | 0.000 | 20 - < 30 years old is lower than 40 - < 50 years old |
| | | | | | 0.000 | 20 - < 30 years old is lower than 50 - 60 years old |
| **Gender** | 0.934 | | 0.000 | Female is higher than male | 0.000 | Female is higher than male |
| **Employment Status** | 0.000 | | 0.000 | | 0.000 | |
| | 0.000 | Student is lower than Teaching Staff | 0.000 | Student is lower than Teaching Staff | 0.000 | Student is lower than Teaching Staff |
| | 0.000 | Student is lower than Administrative Staff | 0.000 | Student is lower than Administrative Staff | 0.000 | Student is lower than Administrative Staff |
| **Place of residence** | 0.000 | Urban is higher than rural areas | 0.924 | | 0.677 | |
| **Level of education***** | 0.000 | | 0.000 | | 0.069 | |
| | 0.000 | Bachelor is lower than PhD | 0.001 | Master is lower than Bachelor | | |
| | 0.000 | Master is lower than PhD | | | | |
| **Have you ever attended COVID-** | 0.000 | Yes is higher than no | 0.599 | | 0.06 | |

| | | | | | | |
|---|---|---|---|---|---|---|
| **19 infection training or orientation session? (yes)** | | | | | | |
| **Have you been infected with COVID-19?(yes)** | 0.000 | Yes is higher than no | 0.000 | No is higher than yes | 0.002 | No is higher than yes |
| **Has any of your colleagues been infected with COVID-19? (yes)** | 0.000 | Yes is higher than no | 0.785 | | 0.808 | |

*Significance for comparison performed by the Mann-Whitney U test (to compare 2 groups) or the Kruskal-Wallis test (to compare >2 groups). This is adjusted p-value after Bonferroni correction.

**In post hoc analysis (in Kruskal-Wallis test), only the significant findings were presented.

***those who answers this question with "other" have been excluded
